# A Bayesian Quantile Modeling for Spatiotemporal Relative Risk: An Application to Adverse Risk Detection of Respiratory Diseases in South Carolina, USA

**DOI:** 10.3390/ijerph15092042

**Published:** 2018-09-18

**Authors:** Chawarat Rotejanaprasert, Andrew B. Lawson

**Affiliations:** 1 Department of Tropical Hygiene, Faculty of Tropical Medicine, Mahidol University, Bangkok 10400, Thailand; 2 Department of Public Health Sciences, Medical University of South Carolina, Charleston, SC 29425, USA; lawsonab@musc.edu

**Keywords:** quantile modeling, spatiotemporal, log Laplace, Bayesian, adverse risk detection, respiratory disease

## Abstract

Quantile modeling has been seen as an alternative and useful complement to ordinary regression mainly focusing on the mean. To directly apply quantile modeling to areal data the discrete conditional quantile function of the data can be an issue. Although jittering by adding a small number from a uniform distribution to impose pseudo-continuity has been proposed, the approach can have a great influence on responses with small values. Thus we proposed an alternative to model the quantiles of relative risk for spatiotemporal areal health data within a Bayesian framework using the log-Laplace distribution. A simulation study was conducted to assess the performance of the proposed method and examine whether the model could robustly estimate quantiles of spatiotemporal count data. To perform a test with a real data example, we evaluated the potential application of clustering under the proposed log-Laplace and mean regression. The data were obtained from the total number of emergency room discharges for respiratory conditions, both infectious and non-infectious diseases, in the U.S. state of South Carolina in 2009. From both simulation and case studies, the proposed quantile modeling demonstrated potential for broad applicability in various areas of spatial health studies including anomaly detection.

## 1. Introduction

The ordinary mean regression has been a main analytic approach in epidemiological studies. It is usually assumed in the regression that the covariates have an effect only on the mean of the outcome distribution; other aspects, such as variance or skewness, are not usually considered. While this method offers attractive features such as computationally efficiency and convenience to interpret, it may not be effective in many cases. For instance, a subtle association can be missed such as risk factor effects on the dispersion or the probability of extreme observations. In addition, mean regression is also known to be susceptible to outliers. On the other hand, quantile modeling has been developed to extend the mean regression to model conditional quantiles of the outcome variables [1,2]. Quantile modeling has been seen as an alternative useful complement to ordinary regression, focusing on mean, in which the upper or lower quantiles of the health events of interest may depend on the variables very differently from the mean [3]. Hence the quantile regression technique may provide a more accurate explanation of functional changes than aiming solely on the mean.

There are a wide range of epidemiological applications using quantile estimation (see examples in [4]; Chapter 13 [3]). For example, Bayesian quantile approaches have been proposed to model continuous response variables including longitudinal quantile modeling [5], exponentially tilted empirical likelihood [6], Bayesian parametric spatial and spatiotemporal quantile regression [7,8,9,10]. In general, a common Bayesian method for continuous quantile regression is to maximize the likelihood of an asymmetric Laplace distribution (AL) which is equivalent to minimizing the quantile loss function [11]. For spatial and spatiotemporal data, Waldmann et al. [12] proposed a general framework for Bayesian semiparametric quantile modeling for areal spatial data. The model is also built upon an asymmetric Laplace assumption for the errors and incorporates quantile-specific random effects to accommodate spatial heterogeneity. However, the approach does not account for temporal variation. Lum and Gelfand [13] proposed a related asymmetric Laplace process for point-process spatial data referenced by a set of geographic coordinates. While there are a large body of literature developed for continuous response, in the case of a count variable, however, adopting quantile regression is not obvious due to the non-continuous nature of its cumulative distribution.

An objective of this work is to propose an alternative to model the spatiotemporal quantiles of relative risk within a Bayesian framework. To apply quantile modeling to areal data in general we have to cope with the problem of the conditional quantile function that is not continuous. Jittering proposed by Machado and Silva is a common approach to model quantiles for a discrete response distribution [14]. However, the approach can be unreliable due to the added noise usually generated from a Uniform distribution which can have a great influence on response with small value. Instead in this work we propose to model the quantile of relative risk which is continuous to avoid the discrete nature of the event count itself using an alternative approach. Thus in this paper we developed a computationally tractable spatiotemporal quantile model in a user-friendly computing environment to be accessible for public health practitioners. The next sections provide the theoretical background on quantile estimation and our proposal for areal health data. The proposed model is then compared with the estimates from mean regression via a ground-truth simulation study. A case study of space-time disease cluster detection is also presented to investigate the behaviors of the standard posterior diagnostics compared with our proposed method to demonstrate its applicability potential.

## 2. Materials and Methods

### 2.1. Quantile Modeling for Count Data

The application of quantile modeling has been widely used in epidemiological studies for continuous response. However, when dealing with count data, a primary challenge is that the quantiles of a discrete random variable are not continuous due to the discrete nature of their cumulative distribution functions. Therefore, they cannot be expressed directly as a continuous function of the covariates of interest. A way to overcome this challenge is to smooth the outcome artificially by adding a noise, usually assumed to be uniformly distributed. The general idea of the approach is to replace the count response with a jittered variable so that the conditional quantiles of the transformed variable after noise added are still preserved as in the quantiles of the original outcome [14]. The jittering methods have been adopted in many econometric (see examples [15,16]) and spatial health applications [10]. Although the approach can solve the problem of a non-continuous response distribution, the mean and variance for small values of the response in the transformed variable will be largely affected by the added noise which can result in inaccurate estimation of the conditional quantiles [17]. Therefore, we propose an alternative to model space-time count data based on the log Laplace (LL) distribution.

According to the paper originally done by Koenker and Bassett [1], the distribution of the error term does not need to be specified as it is allowed to take arbitrary form except the constraint that its τ-quantile equals zero. Typically, the quantile prediction is estimated by optimizing the weighted absolute loss function. However, it is challenging to find an optimal solution for a complex model such as with space-time structured random effects and non-linear functions [18]. Hence flexible Bayesian approaches have been widely implemented and a common model of Bayesian quantile regression is applying an AL distribution to the error term. The method aims to find posterior maximums that are equivalent to the estimates yielded from the quantile loss function using the asymmetrically quantile loss function in linear quantile modeling [11]. However, the support of AL variable is on the real line which might not be appropriate for count data. Thus for analyzing space-time count data, we then apply a log Laplace distribution for modeling the areal data instead. The development of the proposed method is provided in the next section.

### 2.2. Quantile Modeling for Spatiotemporal Relative Risk

#### 2.2.1. Log-Laplace Distribution

Log-Laplace models have seen to appear sporadically in the statistical, economical and scientific literature in past years (see examples in [19,20,21]). A log-Laplace density can be represented as a combination of the Pareto and power function distributions [22]. With an additional parameter, the resulting three-parameter log-Laplace distribution, LL(δ,a,b) of relative risk, μ, is given by the probability density function:

(1) $$f(\mu )=\frac{1}{\delta }\frac{ab}{a+b}\{\begin{array}{c} {\left(\frac{\mu }{\delta }\right)}^{b-1},\;0<\mu <\delta \\ {\left(\frac{\delta }{\mu }\right)}^{a+1},\;\mu \geq \delta \end{array}$$

where δ,a,b>0. Then, we can re-parameterize Equation (1) as and let $b=\frac{1-\tau }{\sigma }$ and $a=\frac{\tau }{\sigma }$. Hence, the probability density function of LL($\mu_{\tau },\tau ,\sigma$) can be re-written (please see details in Supplementary Material S1) as:

(2) $$f(\mu ;\mu_{\tau },\tau ,\sigma )=\frac{1}{\mu }\frac{\tau (1-\tau )}{\sigma }\{\begin{array}{c} \exp\left(-(1-\tau )\frac{\left|\log(\mu )-\mu_{\tau }\right|}{\sigma }\right),\;\log(\mu )<\mu_{\tau }\\ \exp\left(-\tau \frac{\left|\log(\mu )-\mu_{\tau }\right|}{\sigma }\right),\;\log(\mu )\geq \mu_{\tau } \end{array}$$

where σ is the variance, $\mu_{\tau }$ is the mean of relative risk at the τth quantile level. However, we can see that minimizing of the quantile loss function (Supplementary Material S2) is identically equivalent to maximizing of the re-parameterized log-Laplace distribution in Equation (2) above. Hence we propose to use the log-Laplace distribution to directly model the τth quantile of relative risk for areal count data.

Then we redefine the notation as $y_{it}$ being the number of cases observed in area *i* and time *t*. To model the conditional quantiles, the relative risk whose cumulative distribution function is continuous can be modeled instead of the count itself using the log Laplace density. A generic structure of the spatiotemporal model for areal health data can be specified via a Poisson likelihood as $y_{it}\sim Poisson(e_{it}\mu_{it})$ by assuming $\mu_{it}\sim LL(\mu_{\tau_{it},it},\tau_{i},\sigma_{\tau_{i}})$ where the quantile level is usually pre-defined to have a common value across spatial and temporal units as $\tau_{it}=\tau \;\forall i,t$. $e_{it}$ is the expected rate, $\mu_{it}$ is the mean relative risk, and $\mu_{\tau ,it}$ is the τth—quantile relative risk for area *i* and time *t*. Note that the expected rates can be seen as the offset in Poisson models. To estimate the rates, a standard practice is to adopt the concept of indirect standardization (same as in epidemiology) which is the most common form of adjustment for population in each location in which the disease outcomes are compared.

#### 2.2.2. Spatiotemporal Relative Risk Quantile Modeling

To model the τth quantile relative risk, the predictor $\eta_{\tau ,it}$ is decomposed as a linear combination of covariates and space-time random effects as $\eta_{\tau ,it}=X_{it}^{T}\beta_{\tau }+\xi_{\tau ,i}+\lambda_{\tau ,t}+\theta_{\tau ,it}$ where $X_{it}$ is a design matrix of area-level predictors; $\beta_{\tau }$ is a vector of regression coefficients for the specified quantile; $\xi_{\tau ,i}$ and $\lambda_{\tau ,t}$ are the conditional quantile spatial and temporal random effects of area *i* and time period *t*, respectively; and $\theta_{\tau ,it}$ denotes a corresponding conditional quantile space-time interaction. To estimate the quantile parameters, a fully Bayesian framework is adopted in which for all parameters in the model a prior distribution needs to be specified.

We structure the model by borrowing information across neighboring regions and time periods to incorporate spatiotemporal smoothing. The convolution model is modeled to the quantile-specific spatial random effect as $\xi_{\tau ,i}=u_{\tau ,i}+v_{\tau ,i}$ where $u_{\tau ,i}$ and $v_{\tau ,i}$ are employed to capture spatially correlated and unstructured extra variation in the model. It is often important to include both structured and unstructured random effects in a spatial analysis since without prior knowledge unobserved confounding can take various forms. The uncorrelated random effect is described by a zero mean Gaussian prior distribution with variance $\sigma_{\tau ,v}^{2}$. The spatially correlated effect is assumed to have the intrinsic conditional autoregressive model (ICAR) model proposed by Besag et al. [23]. That is, conditionally, $u_{\tau ,i}|u_{\tau ,-i}\sim N\left({\bar{u}}_{\tau ,\Omega_{i}},\sigma_{\tau ,u}^{2}/n_{\delta_{i}}\right)$ where $u_{-i}$ is the vector containing the correlated effect of all regions except the *i*th area. $\Omega_{i}$, $n_{\delta_{i}}$ and ${\bar{u}}_{\tau ,\delta_{i}}$ are a set of spatial neighbors, cardinality and the mean of the neighborhood of the *i*th tract respectively, and $\sigma_{\tau ,u}^{2}$ is the spatial component variance. Notably there are a number of models can be specified for these spatial random effects, including simultaneous autoregressive (SAR) or geostatistical models. Among those models, CAR priors are perhaps the most common practice in areal data mapping. A number of globally smooth CAR priors have been proposed, and a review and comparison can be found in [24].

To capture the temporal trend a random walk model is adopted. In general, a random walk is assumed to have a prior distribution as a Gaussian distribution with mean the previous time point which can be both positive and negative. Then the temporal trend can be expressed as $\lambda_{\tau ,t\;i}\sim N\left(\lambda_{\tau ,t-1},\sigma_{\tau ,\lambda }^{2}\right)$ which allows for a type of non-parametric temporal trend effect. The space-time interaction random effect, $\theta_{\tau ,it}$, is specified by a Gaussian distribution with zero mean and variance $\sigma_{\tau ,\theta }^{2}$. All variances in Gaussian prior distributions are described by a relatively non-informative Uniform distribution as $\sigma_{\tau ,*}\sim Unif(0,10)$, where $\sigma_{\tau ,*}=\sigma_{\tau ,u},\sigma_{\tau ,v},\sigma_{\tau ,\lambda },\sigma_{\tau ,\theta }$.

Hence incorporating this stochastic representation of quantile error term and random effects, our proposed model becomes:

(3) $$\begin{array}{l} y_{it}\sim Poisson(e_{it}\mu_{it})\\ \log(\mu_{it})=\log(\mu_{\tau ,it})+\varepsilon_{\tau ,it}\\ \log(\mu_{\tau ,it})=\eta_{\tau ,it}=X_{it}^{T}\beta_{\tau }+\xi_{\tau ,i}+\lambda_{\tau ,t}+\theta_{\tau ,it}. \end{array}$$

Note that the detail of quantile error, $\varepsilon_{\tau ,it}$, approximation using log-Laplace distribution in Equation (3) is provided in Supplementary Material S3.

An aim of this study is not only to theoretically develop a generic and robust methodology for spatiotemporally varying quantile-specific estimation for areal health data, it is also of practical importance to consider a computationally feasible and robust methodology to implement in user-friendly environment. The proposed framework provides accessibility to a wide range of practitioners especially in public health applications as the model specification can be conveniently implemented in standard software such as R or BUGS. Thus not only our method has theoretical foundation but also offers practical flexibility to non-technical users. Then a simulation study with ground-truth scenarios was carried out to demonstrate the performance of the approach.

### 2.3. Simulation Study

A traditional way to estimate the τth quantiles of spatiotemporal count data is using the posterior distribution produced from mean regression. By the mean regression here we imply to the model that the τth quantiles are estimates from the posterior quantiles in the converged samplers from fitting the conventional model without assuming a density for the error quantiles. A simulation study was conducted to assess how the mean and proposed quantile models could robustly estimate quantiles of spatiotemporal count data. For an unbiased evaluation, a non-standard form of the simulated error distribution was assumed to neither be Gaussian nor Laplace distributions because assuming the error to have a Laplace distribution could lead to overly optimistic estimation and assuming a Normal density would imply the conventional regression.

The map used in this simulation study was the state of South Carolina (SC), USA, which has 46 counties and the time periods were assumed to be yearly over a 5-year period. The expected counts were calculated from real data, the total number of emergency room discharges (ERD) for respiratory conditions, both infectious and non-infectious diseases, in SC in 2009 and assumed to be constant over the time periods. Note that we would like the simulation study to reflect some features from a real situation in which the expected rates are obtained, and calculation details of the expected rate will be provided later in the case study. Let the observed incidence for county *i* and year *t* be a Poisson random variable as $y_{it}\sim Poisson(e_{i}\mu_{it})$. The relative risk is set to link with the logarithm function as $\log(\mu_{it})=\eta_{it}+\varepsilon_{\tau ,it}$. The errors were generated from a *t*-distribution with zero center and variance $\sigma_{\varepsilon ,it}^{2}=1$ with the degree of freedom as $e_{i}$ in Equation (4), i.e., $\varepsilon_{it}\sim t_{e_{i}}(0,1)$. The pseudocode is provided in the Supplementary Material S4 for details of the simulated data. The expected number (please see more calculation details in the case study) can be seen as the offset in Poisson models. To estimate the rates, a standard practice is to adopt the concept of indirect standardization (same as in epidemiology) which is the most common form of adjustment for population in each location in which the disease outcomes are compared. The number of expected rate in each location can generally be calculated as the average disease rate for the whole study region multiplied by the population at risk at each location. For further information about calculation of standardized rate, please refer more to, for example, [25,26].

Without covariates, the linear predictor linked to random effects was specified as $\eta_{it}=u_{i}+v_{i}+\lambda_{t}+\theta_{it}$. With the intercept assumed to be zero, the spatial components, $u_{i}$ and $v_{i}$ respectively, were specified to have an ICAR and zero-mean Gaussian models with variances of 1 and 0.2 respectively. The temporal random effect, $\lambda_{t}$, was modeled using the random walk prior with variance of 0.2 and generated from a Normal distribution with zero mean at *t* = 1. The interaction term, $\theta_{it}$, was generated by a zero-mean Gaussian prior with variance of 0.2. It should be noted that the random effects and error terms were simulated on the log scale. Thus, although the variances used in simulation seem to be small (on log scale), the middle 80% of the generated relative risks is on the range of 0.12 and 9.07. Although the simulated risks seem to be extreme, there was evidence of high relative risk in some areas from the real data used later in case study section. For example, the relative risk in Greenwood county (please see in the case study Section) had reached the level of 8 during a peak period. Hence the parameter setting was chosen so that the extreme behavior was captured in the simulation study.

An aim of the simulation study is to evaluate whether the proposed model can well predict the true quantiles as the fitting under a log-Laplace distribution is not assumed. Since we assume a symmetric zero-mean distribution for the errors, the error median is zero and hence the simulated log relative risk is determined entirely by the linear predictor, $\eta_{it}$, at median, i.e., $Q_{0.5}(\mu_{it})=\exp(\eta_{it})$. For other τ quantile levels, the simulated $Q_{\tau }(\mu_{it})$ is driven by both the random effect and error terms. Therefore, the true model was generated from non-Laplace likelihood.

To compare the performance of the proposed quantile and mean models, fifty simulated data sets were generated and the regression models were fitted at the quantile levels of 0.9, 0.5, and 0.1. For each scenario, the estimated quantiles from log-Laplace and mean models were compared with the true simulated values. The estimated quantiles from the mean model were obtained at the τth level out of the converged posterior samplers with the same prior specification as the quantile model while the estimated quantiles from the proposed quantile model were calculated under the log-Laplace model as ${\hat{Q}}_{\tau }(\mu_{it})=\exp({\hat{\eta }}_{\tau ,it})$ since $Q_{\tau }(\varepsilon_{it})=0$. The simulation was conducted using WinBUGS software. Each model was run for 50,000 MCMC iterations with a burn-in period of 50,000 samplers and the results were from 100 simulations. To assess the mixing of posterior samplers, we adopted Gelman’s $\hat{R}$ statistics proposed in [27,28] for multiple chain convergence. Various MCMC methods are implemented in WinBUGS and defaults algorithms are used depending on the hierarchical structure in each part of the model such as direct sampling using standard algorithms and slice samplings [29]. A WinBUGS code for quantile estimation is provided in Supplementary Material S5.

**Figure 1:**
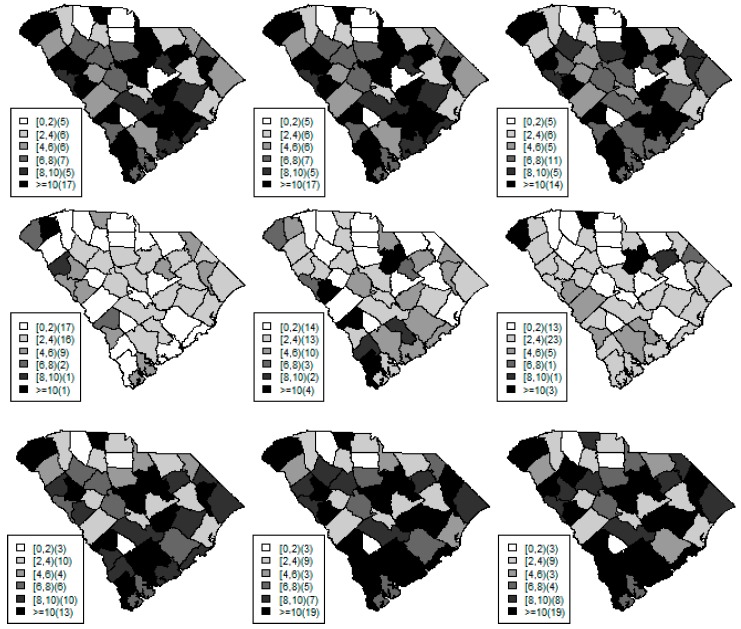
Maps of the estimated 90th quantile relative risks from the proposed quantile (**top** row) and mean (**middle** row) regression models versus the simulated relative risks (**bottom** row) during years 2 (left) to 4 (right).

**Figure 2:**
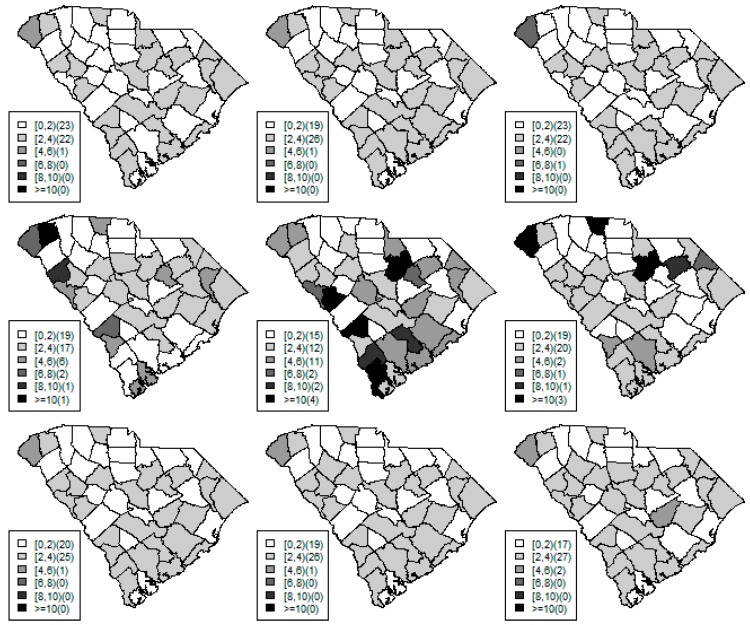
Maps of the estimated median quantile relative risks from the proposed quantile (**top** row) and mean (**middle** row) regression models versus the simulated relative risks (**bottom** row) during years 2 (left) to 4 (right).

**Figure 3:**
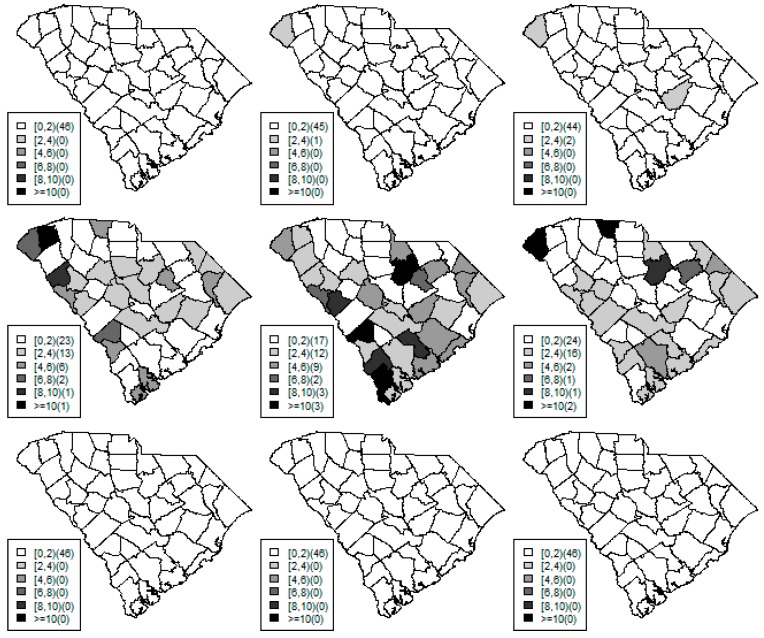
Maps of the estimated 10th quantile relative risks from the proposed quantile (**top** row) and mean (**middle** row) regression models versus the simulated relative risks (**bottom** row) during years 2 (left) to 4 (right).

Figure 1, Figure 2 and Figure 3 display the true and estimated relative risk quantiles averaged over simulated data sets at 90th, 50th, and 10th respectively for each county during years 2–4. The upper and middle rows show the estimated quantiles from the proposed quantile and conventional mean regression models whereas the bottom row displays the true relative risks for each area and time period. 

The estimated and true space-time relative risks are similar at the quantile levels under log Laplace regression while mean regression appears to underestimate the true 90th quantiles and overestimate the 10th quantiles. Importantly, from Figure 2, our proposed model seems to produce space-time quantile estimates more similar to the simulated than the ones from the mean regression model even at the 50th quantile level. This suggests that our proposed model can reliably estimate extreme quantiles and perhaps even more robustly predict medians than the mean regression across the space-time units although the error density was not assumed by the fitted model.

**Figure 4:**
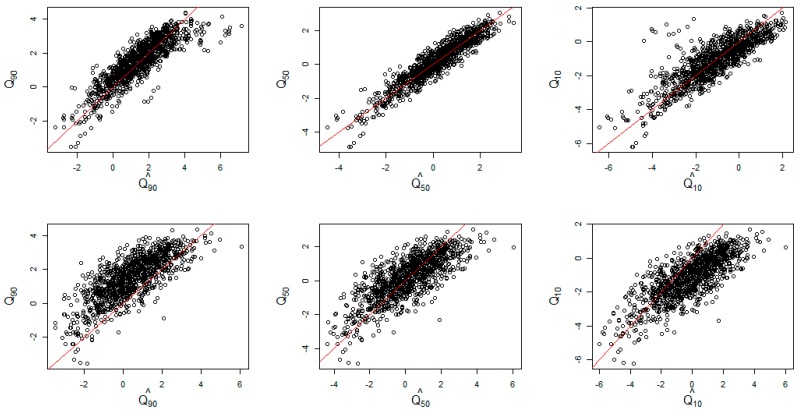
Scatterplots of simulated and estimated from quantile (**upper**) versus mean (**lower**) regression models at 10th (left), 50th (middle), and 90th (right) quantiles on the log scale. The red line represents the equality of the predicted and true quantiles.

Figure 4 displays the scatterplots of simulated and estimated from quantile (upper) versus mean (lower) regression models at 10th (left), 50th (middle), and 90th (right) quantiles. The red line represents the equality of the predicted and true quantiles. Both the log Laplace and mean regression appear to provide the most accurate estimates of spatiotemporal quantiles at τ=0.5. To evaluate the models quantitatively, the mean squared error (MSE) were compared as shown in Table 1. The MSE at the 50th quantile level under the proposed regression was almost six times less than that under the mean model.

The difference of the log-Laplace and mean models were further apparent at more extreme quantile levels. At τ= 0.9 and 0.1, the MSEs from our proposed model were much smaller than the ones from mean regression. Also as seen from Figure 4, the predicted values from the log-Laplace regression (upper row) were closer to the red line with smaller variation than the mean regression (lower row) at both τ= 0.9 and 0.1. This supports the earlier result that the mean model tends to overestimate at the low quantile levels and underestimate the true values at the high quantile levels. From these findings it appears show that the more accurate quantile estimates for spatiotemporal areal data can be achieved by our proposed modeling strategy opposed to the conventional mean model, even though the error distributional assumptions fail or even at the central quantile.

## 3. A Case Study and Results

### 3.1. Posterior Estimates in Adverse Risk Detection

Quantile models have been developed and used in spatiotemporal health applications. A primary application in spatial epidemiology is to determine the relationship between the covariate risk factors and the conditional quantile of the distribution of a dependent variable. However, besides studying the relationship between the covariates and relative risk of outcome, in the study of disease spatial distribution, it is often appropriate to ask questions related to the local properties of the relative risk surface. Local properties of the surface could include peaks or clusters of risk, sharp boundaries between areas of risk, or local heterogeneities in risk. There are a variety of definitions of clusters and clustering. Here a disease cluster is considered as any group of areas displaying “usual” risk and an isolated area with excess risk is referred as a hot spot. Testing based methods, such as scan statistics, have been applied in the context of cluster detection. Nonetheless, limitations of testing based approaches lie on the restrictive hypotheses testing, geometric search regions and their inability to incorporate both fixed and random effect modifiers. Thus only modeling based cluster methods are considered here.

A standard modeling based approach to cluster detection is to consider measures of quantities monitored in the posterior distribution that may contain clustering information. Perhaps the most commonly used instance of these measures is the exceedence probability proposed by Richardson et al. [30]. The exceedence probability which can be estimated by recording how often the relative risk exceeds a threshold has been used to evaluate how unusual the risk is in an area. The exceedence probability of the relative risk for each area and time period is usually calculated from the posterior sample values and defined as:

$$P(\mu_{it}\geq c)\approx \hat{P}(\mu_{it}\geq c)=\sum_{g}^{G}I(\mu_{it}^{g}\geq c)/G$$

where *I* is the indicator function and *g* indicates the posterior samplers after the burn-in period. There are two components needed to calculate the probability: the cut-off point *c* and the threshold for a probability to define an unusual risk. Some thresholds conventionally chosen are, for example, 0.95, 0.975, and 0.99 while *c* can be a range of extreme risks such as 1 or 2 and 3. Larger values of *c* represent more extreme risk levels.

The idea of exceedence probability was proposed as simple mapping the posterior mean relative risk does not make comprehensive use of the outcome of the Bayesian analysis that makes available samples from the whole posterior distribution. However, the use of exceedence probability depends on the method that has been fitted to. From the previous section, the result of simulation study suggests that the mean regression potentially produces narrower credible intervals, i.e., underestimates the posterior variance. This could have a large effect on estimating the exceedence probability. Therefore, we aim to evaluate the tail probability yielded from the quantile and mean regression, and demonstrate another potential application of our proposed modeling strategy in the context of adverse risk detection which is yet to be investigated using quantile modeling.

### 3.2. A Case Study of Adverse Risk Detection of Respiratory Diseases in South Carolina, USA

**Figure 5:**
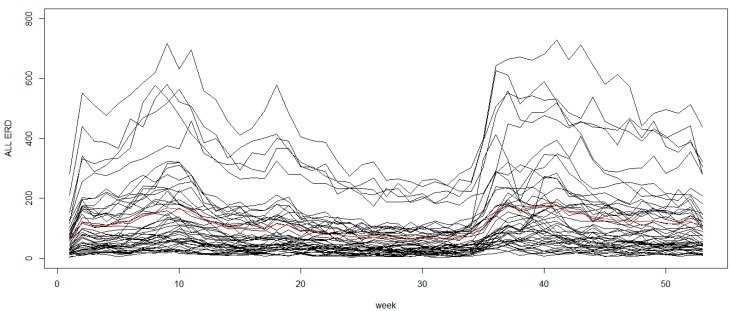
Weekly emergency room discharges of respiratory diseases in South Carolina 2009. The red line represents the mean number of cases averaged over all counties.

We compare the performance of the exceedence probability under the quantile and mean regression using a real data example. The data are from the total number of emergency room discharges (ERD) for respiratory conditions, both infectious and non-infectious diseases, in the state of South Carolina (SC) in 2009. The data include acute upper respiratory infections, influenza, acute bronchitis, asthma, emphysema and pneumonia obtained by county for the 46 counties from the South Carolina Office of Research and Statistics. While these diseases can occur in the population at any season, they commonly happen during the fall and winter periods (see Figure 5). We used the total number of all conditions combined to find overall anomalies. Although we demonstrate with the case study of combined conditions, the proposed methods can also be applied to individual or a group of diseases with shared etiology.

Because of the lack of a standard population with age-specific rate for the disease, the internal data standardization during the endemic period was adopted to calculate the expected count for each county. We disregarded the data from week 1 to week 25 as the history in the previous epidemic period, and used the information beginning from week 26 until week 31 to calculate the expected rate because it was assumed to be the endemic period. The expected rate for each region i and time t was computed as:

(4) $$e_{i}=pop_{i}\times \frac{\sum_{i}\sum_{t\in T_{en}}ERD_{it}/|T_{en}|}{\sum_{i}pop_{i}}$$

where $pop_{i}$ is the average number of inhabitants per month in SC in 2009 and $ERD_{it}$ is the all weekly number of ERD cases in the county i and time t. $T_{en}=\{26,\ldots ,31\}$ is the duration of the endemic with size of $\left|T_{en}\right|$ = 6 days. Note that for the calculation of expected rates, when covariates are not available, the value of expected rate in each location can be calculated as the average disease rate for the whole study region multiplied by the population at risk at each location. For further information about calculation of standardized rate, please refer to for example [25,26]. The expected rates in Equation (4) were also assumed to be constant over time, a sensible conclusion because we used the information from the endemic period to calculate the expected rates representing the background variation under the null situation.

According to Koenker and Bassett [1], the distribution of the error term does not need to be pre-specified besides the constraint that the estimate need to satisfies the quantile loss function with its τth quantile equals zero, i.e., $F_{\varepsilon_{it}}\left(0|\mu_{it}\right)=\tau$ or $Q_{\tau }(\varepsilon_{it})=0$. So for a constant *c*, which represents the null values of relative risk usually assumed to be 1 (null situation) or 2 (high relative risk), we can construct the τth quantile relative risk compared to the null values of interest as $\log(c)=\log(\mu_{it})+\varepsilon_{\tau ,it}$. The error term is the deviation of the relative risk to the reference value *c* on the log scale. From the constraints of quantile regression, we have that:

(5) $$\begin{array}{l} \tau =F_{\varepsilon_{it}}\left(0|\mu_{it}\right)\\ ↔P(\varepsilon_{\tau ,it}\leq 0)\\ ↔P(\varepsilon_{\tau ,it}+\log(\mu_{it})\leq \log(\mu_{it}))\\ ↔P(\log(c)\leq \log(\mu_{it}))\\ ↔P(c\leq \mu_{it}). \end{array}$$

Hence, in Equation (5) we have that the exceedence probability estimated using quantile regression is in fact $F_{\varepsilon_{it}}\left(0|\mu_{it}\right)=P(c\leq \mu_{it})=\tau$. That is we can alternatively compute the exceedence probability by estimating the quantile level from setting $\log(c)=\log(\mu_{it})+\varepsilon_{\tau ,it}$. The threshold needs to be pre-specified, which is normally set to be one to represent a null situation, or two for a more extreme condition. To compute the posterior distribution of the quantile level τ, we can set that $\log(c=1\;or\;2)=\log(\mu_{it})+\varepsilon_{\tau ,it}$ by assuming Beta(1,1) for τ. Other hyper-parameters for $\mu_{it}$ and $\varepsilon_{\tau ,it}$ can be modeled as specified in the previous section. 

**Figure 6:**
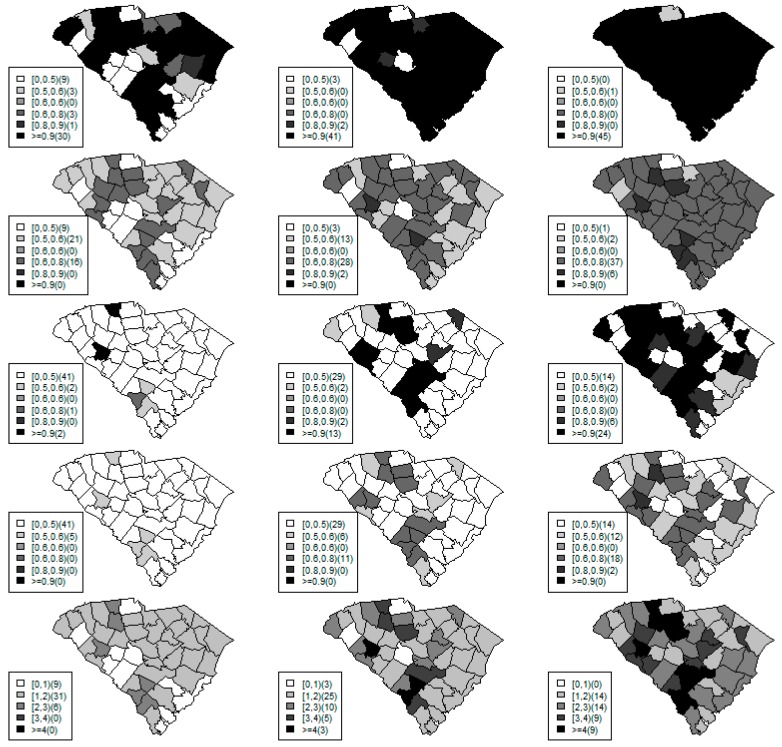
Maps of exceedence probability under mean regression for each area with cut-off points of 1 (*c* = 1; first row) and 2 (*c* = 2; third row). Maps of exceedance probability under quantile modeling with cut-off points of 1 (*c* = 1; second row) and 2 (*c* = 2; forth row) compared to the posterior estimated relative risk (fifth row) during weeks of 34 (left column), 35 (middle), and 36 (right column).

Figure 6 shows the exceedence probabilities estimated from the mean and quantile regression during the weeks of 34–36 with the cut-off points (*c*) of 1 and 2, compared to the posterior estimated relative risk. At *c* = 1, the values of exceedence probability under the mean regression (Figure 6; row 1) are much higher than the one under quantile modeling across areas and time periods. This also happens at *c* = 2. At week 36 when there are potential outbreaks in some counties (see Figure 6; row 5), the mean model gives a high alarm for almost all counties while the quantile estimation provides more sensible signals at both thresholds levels of 1 and 2. The maps of exceedence probability with threshold of one (first row in Figure 6) show that the mean regression tends to overestimate the tail probability with the null baseline (threshold of one) while it indicates hot spots in week 33 and larger clusters with strong alarms throughout the weeks.

**Figure 7:**
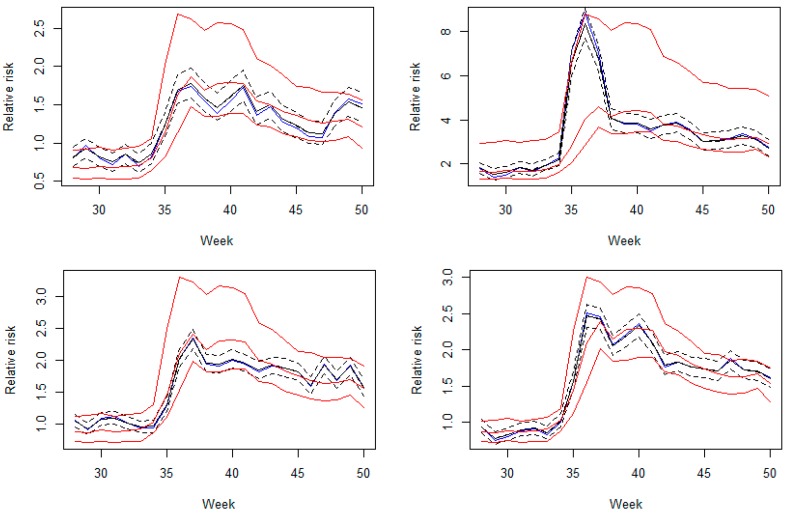
Plots of crude relative risks (standardized incidence rate; blue), posterior mean relative risks (solid black) with corresponding 95% credible intervals (dash black), and estimated 5th, 50th and 95th quantiles from log-Laplace regression (solid red) for Beaufort (upper left), Greenwood (upper right), Charleston (lower left), and Richland (lower right) counties.

Figure 7 displays the plots of crude (standardized incidence rate, SIR), posterior mean, and posterior median relative risks with corresponding 90% credible intervals of four counties representing both rural and urban areas. All credible intervals from the quantile model are much wider when a jump occurs which is sensible as it has to account for extreme observations while it appears that mean regression yields much smaller and symmetric credible intervals than the ones from quantile modeling. This result shows a significant example of cluster evaluation that mean regression can send a strong alarm even when there is only a tiny shift in relative risk. This is consistent with the result from the exceedence probability in Figure 6 that mean regression could potentially produce more false positives at the same threshold for an exceedence probability to define an unusual risk.

Also, at week 35 in Beaufort county (upper left panel in Figure 7), the mean regression would suggest an adverse risk since almost 100% of the 95% credible band is above the level of one although the posterior mean is only slightly larger than one. In contrast the exceedence probability computed from the proposed quantile estimation would yield a moderate value of exceedence probability at the threshold of one (second row in Figure 6). With the threshold of two, the results from our method perhaps more reasonably suggests possible clusters/hotspots with a similar pattern as the estimated posterior relative risk (last row in Figure 6) than the ones from the mean regression (third row in Figure 6) which mainly gives extremely high tail probabilities in most areas. The strong tail probabilities could be resulted from the small posterior variance in the relative risk under the mean regression. It should be noted that another interesting characteristic of exceedence probability under mean regression is that its credible bands appear to be almost always symmetric.

## 4. Discussion

As a result of this paper several interesting modeling extensions are possible to be further applied and investigated. We have demonstrated potential use of our proposed methodology in the simulation study and case study, and provided the relationship between exceedence probability and estimated quantile level which can be used as a measure of extreme observations. In general, a Poisson likelihood with random effects (both spatial and non-spatial components) has been considered as a standard tool in Bayesian disease mapping to cope with extra variation in both likelihood specification (overdispersion) and unobserved confounders [23,31,32,33]. However, the results from both simulation study and data example suggest that the model may be insufficient to deal with extra-Poisson variability. Due to initial simulation study in this paper, an area of future work is possible to further examine other model specifications or distributions that could efficiently accommodate the extra variation [34]. The proposed method can also be extended to other modeling such as negative binomial and zero-inflation models which are alternatives for coping with dispersion issue.

## 5. Conclusions

In this paper we have proposed an alternative to model the quantiles of spatiotemporal relative risk. Not only do we provide theoretical justification of forming quantile estimation based on the log-Laplace distribution, the approach also offers computational convenience for public health practitioners using standard software. A simulation study was conducted to assess the performance of the proposed model and examine if the model could robustly estimate quantiles of spatiotemporal count data. For an unbiased evaluation, a non-standard form of the simulated error distribution was assumed to neither be Gaussian nor Laplace distributions. The simulation results show that the mean model tends to overestimate at the low quantile levels and underestimate the true values at the high levels while more accurate quantile estimates for spatiotemporal areal data can be achieved by our proposed modeling strategy even when the distributional assumption does not hold.

We applied the proposed quantile modeling in the field of surveillance that is an area in which this methodology is yet to be used. A standard modeling based practice to cluster detection is the exceedence probability. We evaluated the performance of the exceedence probability under the proposed quantile and mean regression using a real data example. The data are from the total number of emergency room discharges for respiratory conditions, both infectious and non-infectious diseases, in the state of South Carolina in 2009. Under mean regression, the exceedence probability mostly gives extremely high tail probabilities in most areas. The high tail probabilities from mean regression could well be a result from the narrow posterior bands of relative risks while our proposed quantile model gives perhaps more sensible exceedence maps comparing to the posterior relative risk estimates. Interestingly, we also found that the credible bands appear to be almost always symmetric under mean regression. As seen, the modeling strategy developed in this paper is not only having theoretical foundation with convenient computation, but also has potential for broad applicability in various areas of spatial health studies.

## Figures and Tables

**Table 1:** Mean squared error (MSE) from proposed quantile and mean regression models.

Level	0.9	0.5	0.1
Quantile model	689.54	236.64	742.08
Mean model	2446.15	1245.37	2734.15

## Supplementary Materials

The following are available online at http://www.mdpi.com/1660-4601/15/9/2042/s1.
