# Supplementary material for: A Bayesian Quantile Modeling for Spatiotemporal Relative Risk: An Application to Adverse Risk Detection of Respiratory Diseases in South Carolina, USA
Source: Int J Environ Res Public Health. 2018 Sep 18;15(9):2042. doi: 10.3390/ijerph15092042 (PMC6164988; doi:10.3390/ijerph15092042)
Supplement: Supplementary file 1 [file ijerph-15-02042-s001.pdf]

Supplementary Materials

S1) Re-parametrization of log-Laplace distribution

The three-parameter log-Laplace distribution, LL(δ, a, b) of relative risk, μ, is given by the probability density function

$$f(\mu)=\frac{1}{\delta}\frac{ab}{a+b}\begin{cases}\left(\frac{\mu}{\delta}\right)^{b-1}, 0<\mu<\delta \\ \left(\frac{\delta}{\mu}\right)^{a+1}, \mu\geq\delta\end{cases}.$$

Let $b=\frac{1-\tau}{\sigma}$ and $a=\frac{\tau}{\sigma}$. Hence, the probability density function of LL(μ_τ, τ, σ) can be re-written as

$$\begin{aligned}f(\mu)&=\frac{1}{y}\frac{ab}{a+b}\begin{cases}\left(\frac{\mu}{\delta}\right)^{b}, 0<\mu<\delta=\exp(\mu) \\ \left(\frac{\delta}{\mu}\right)^{a}, \mu\geq\delta=\exp(\mu)\end{cases}\\
&=\frac{1}{\mu}\frac{ab}{a+b}\begin{cases}\exp(b(\log(\mu)-\mu)), \ \log(\mu)<\mu \\ \exp(a(\mu-\log(\mu))), \ \log(\mu)\geq\mu\end{cases}\\
&=\frac{1}{\mu}\frac{ab}{a+b}\begin{cases}\exp(-b\,|\log(\mu)-\mu|), \ \log(\mu)<\mu \\ \exp(-a\,|\mu-\log(\mu)|), \ \log(\mu)\geq\mu\end{cases}\end{aligned}$$

$$=\frac{1}{\mu}\frac{\tau(1-\tau)}{\sigma}\begin{cases}\exp\left(-(1-\tau)\frac{|\log(\mu)-\mu_\tau|}{\sigma}\right), \ \log(\mu)<\mu_\tau \\ \exp\left(-\tau\frac{|\log(\mu)-\mu_\tau|}{\sigma}\right), \ \log(\mu)\geq\mu_\tau.\end{cases}$$

S2) Bayesian quantile estimation

Let Y_i be the continuous response variable i and $\mathbf{X}_i$ be the corresponding vector of covariates with the first element equals one. At a given quantile level $\tau\in(0,1)$, the τ th conditional quantile of y_i given $\mathbf{X}_i$ is then $Q_\tau(Y_i\,|\,\mathbf{X}_i)=\mathbf{X}_i^T\boldsymbol{\beta}_i(\tau)$ where $Q_\tau(Y_i\,|\,\mathbf{X}_i)$ is the τ th conditional quantile and $\boldsymbol{\beta}_i(\tau)$ is a vector of quantile coefficients. Contrary to the mean regression generally aiming to minimize the squared loss function, the quantile regression links to a special class of check or loss function. The τ th conditional quantile can be estimated by any solution, $\hat{\boldsymbol{\beta}}_i(\tau)$, such that $\hat{\boldsymbol{\beta}}_i(\tau)=\arg\min\sum_i\rho_\tau(Y_i-\mathbf{X}_i^T\boldsymbol{\beta}_i(\tau))$ where $\rho_\tau(z)=z(\tau-I(z<0))$ is the quantile loss function given in (1) and $I(.)$ is the indicator function.

The use of independently distributed asymmetric Laplace distributions was proposed by Koenker and Machado (2) directly related to the optimization problem in the quantile loss function using likelihood-based inference. Then a Bayesian approach to quantile regression was introduced by Yu and Moyeed (3). Notably, however, that there were some other proposed Bayesian methods

but they mostly focused on median rather than full quantile levels. An approach based on a scale mixture of Gaussian variables was suggested by Tsionas (4), which also leads to an AL distribution. Other approaches are based on Bayesian non-parametric priors (5, 6) or substitution likelihoods (7). Quantile modeling has been adopted in the context of longitudinal studies (8-10). However, the semiparametric approaches for quantile modeling require composite structures of prior distributions and hyper parameters. The distinct advantages of Bayesian quantile regression based on the AL distribution were demonstrated in Hewson and Yu (11) and Yu and Stander (12). The AL can be re-parameterized in many forms (see Kotz *et al.* (13)). Yu and Zhang (14) proposed a form AL distribution characterized by three parameters of location, precision, and skewness parameters, (μ, σ and τ respectively) which can be applied directly to model the quantile of interest as $L(\boldsymbol{\beta}(\tau), \sigma \mid \boldsymbol{Y}) = \prod_i \frac{\tau(1-\tau)}{\sigma} \exp\left(-\rho_\tau\left(\frac{y_i - \mu_i(\tau)}{\sigma}\right)\right)$. The kernel of the AL is proportional to the quantile loss function. Thus minimizing the quantile loss function is equivalent to the optimization of the working likelihood using the AL error (3).

S3) Log-Laplace approximation to quantile error

The log-Laplace distribution can be derived in forms of other commonly known distributions, such as the Pareto, exponential, lognormal, and beta distributions. The log-Laplace density $LL(\delta, a, b)$ can be also seen as a mixture of lognormal distribution $LN(\mu, \sigma)$. This is a result of the fact that an AL random variable can be viewed as a normal variable (13). More specifically, the relative risk following the log Laplace distribution has a representation as $\mu = \exp(\zeta) R^{\sigma}$ in distribution where $R \sim LN(0,1)$, $\zeta = \log(\delta) + \left(\frac{1}{a} - \frac{1}{b}\right)\psi$ and $\sigma = \sqrt{\frac{2\psi}{ab}}$, where $\psi \sim Exp(1)$ and independent of R (15). However, the asymmetric Laplace and log Laplace random variables can be viewed as normal and log normal random variables respectively. So as a direct result, the relative risk can be expressed as $\log(\mu_{it}) = \log(\mu_{\tau,it}) + \varepsilon_{\tau,it}$ where $\mu_{\tau,it}$ is modeled as an additive mixed model linked to the quantile-specific (non) linear predictor, $\eta_{\tau,it}$, as $\log(\mu_{\tau,it}) = \eta_{\tau,it}$. The error term $\varepsilon_{\tau,it}$ is the quantile error term with the τ th quantile is zero, i.e. $p(\varepsilon_{it} \le 0 \mid \eta_{it}) = \tau$ or $Q_\tau(\varepsilon_{it}) = 0$. Then the density of $\varepsilon_{\tau,it}$ can be written as

$$f\left(\varepsilon_{\tau,it}\right) = \frac{\tau(1-\tau)}{\sigma} \exp\left\{-\frac{\varepsilon_{it}[\tau - I(\varepsilon_{it} < 0)]}{\sigma}\right\}$$

which is in a form of asymmetric Laplace distribution. However, as mentioned, the AL distribution can be viewed as a scale mixture of normal random variables, facilitating forms of Gibbs sampling (16), as

$$\varepsilon_{\tau,it} = \frac{1-2\tau}{\tau(1-\tau)}\psi_{it} + \sqrt{\frac{2\psi_{it}}{\omega\tau(1-\tau)}}Z$$

where $\psi_{it} \sim Exp(\omega)$ and $Z \sim N(0,1)$. Conditional on ψ_{it}, the error tern follows a Gaussian distribution, i.e. $\varepsilon_{\tau,it} \sim N\left(\frac{1-2\tau}{\tau(1-\tau)}\psi_{it}, \frac{\delta\tau(1-\tau)}{2\psi_{it}}\right)$. Hence the posterior computation can be conveniently implemented in standard software such as R or BUGS.

S4) Pseudocode for simulated data

for each simulation {

assign sigma = 1
for each location{
generate the non-spatial random effect (v_i) ~ Normal(0,0.2)
generate the spatial random effect (u_i) ~ ICAR(0.2)
}
for time point {
generate the temporal effect (λ_1) ~ Normal (0,0.2) for the first time point

generate the temporal effect (λ_t) ~ Normal (λ_{t-1},0.2) for the next time points

}
for each location and time point{

generate the space-time interaction (θ_{it}) ~ Normal(0,0.2)

generate a random error ~ t-student(0,1,e_i)
generate a τ th-quantile error from the same t-student distribution

generate τ th-relative risk = exp(v_i+u_i+ λ_1 + θ_{it} + random error + τ th-quantile error)

generate τ th-quantile count data as $y_{\tau,it} \sim Poisson(e_{it}\mu_{\tau,it})$

}
}

S5) WinBUGS code for quantile modeling of spatiotemporal relative risk

```
model{
for(i in 1:I){
    for(t in 1:T){
        y[i,t] ~ dpois(m[i,t])
        log(m[i,t]) <- mm[i,t]+qe[i,t]+log(e[i])
        qe[i,t] <- ((1-2*q)/(q*(1-q)))*w[i,t]+sqrt((2*w[i,t])/(tau*q*(1-q)))*z[i,t]
        w[i,t] ~ dexp(tau)
        z[i,t] ~ dnorm(0,1)
        mm[i,t] <- a+v[i]+u[i]+lambda[t]+theta[i,t]
        theta[i,t] ~ dnorm(0,tau.theta)
    }
    v[i] ~ dnorm(0,tau.v)
}
lambda[1] ~ dnorm(0,tau.lambda)
for(t in 2:T){
    lambda[t] ~ dnorm(lambda[t-1],tau.lambda)
}
u[1:I] ~ car.normal(adj[], weights[], num[], tau.u)
for(i in 1:sumNumNeigh){
```

```
        weights[i] <- 1
}
}
```

References

1. Koenker R, Bassett Jr G. Regression quantiles. Econometrica: journal of the Econometric Society. 1978:33-50.

2. Koenker R, Machado JA. Goodness of fit and related inference processes for quantile regression. Journal of the american statistical association. 1999;94(448):1296-310.

3. Yu K, Moyeed RA. Bayesian quantile regression. Statistics & Probability Letters. 2001;54(4):437-47.

4. Tsionas EG. Bayesian quantile inference. Journal of statistical computation and simulation. 2003;73(9):659-74.

5. Kottas A, Gelfand AE. Bayesian semiparametric median regression modeling. Journal of the American Statistical Association. 2001;96(456):1458-68.

6. Kottas A, KRNJAJIĆ M. Bayesian semiparametric modelling in quantile regression. Scandinavian Journal of Statistics. 2009;36(2):297-319.

7. Dunson DB, Taylor JA. Approximate Bayesian inference for quantiles. Nonparametric Statistics. 2005;17(3):385-400.

8. Geraci M, Bottai M. Quantile regression for longitudinal data using the asymmetric Laplace distribution. Biostatistics. 2007;8(1):140-54.

9. Liu Y, Bottai M. Mixed-effects models for conditional quantiles with longitudinal data. The International Journal of Biostatistics. 2009;5(1).

10. Yuan Y, Yin G. Bayesian quantile regression for longitudinal studies with nonignorable missing data. Biometrics. 2010;66(1):105-14.

11. Hewson P, Yu K. Quantile regression for binary performance indicators. Applied Stochastic Models in Business and Industry. 2008;24(5):401-18.

12. Yu K, Stander J. Bayesian analysis of a Tobit quantile regression model. Journal of Econometrics. 2007;137(1):260-76.

13. Kotz S, Kozubowski T, Podgorski K. The Laplace distribution and generalizations: a revisit with applications to communications, economics, engineering, and finance: Springer Science & Business Media; 2001.

14. Yu K, Zhang J. A three-parameter asymmetric Laplace distribution and its extension. Communications in Statistics—Theory and Methods. 2005;34(9-10):1867-79.

15. Kozubowski TJ, Podgórski K. Log-Laplace distributions. International Mathematical Journal. 2003;3:467-95.

16. Yue YR, Rue H. Bayesian inference for additive mixed quantile regression models. Computational Statistics & Data Analysis. 2011;55(1):84-96.
